# Inferring Transmission Bottleneck Size from Viral Sequence Data Using a Novel Haplotype Reconstruction Method

**DOI:** 10.1128/JVI.00014-20

**Published:** 2020-06-16

**Authors:** Mahan Ghafari, Casper K. Lumby, Daniel B. Weissman, Christopher J. R. Illingworth

**Affiliations:** a Department of Genetics, University of Cambridge, Cambridge, United Kingdom; b Department of Physics, Emory University, Atlanta, Georgia, USA; c Department of Zoology, University of Oxford, Oxford, United Kingdom; d Department of Applied Mathematics and Theoretical Physics, University of Cambridge, Cambridge, United Kingdom; e Department of Computer Science, Institute of Biotechnology, University of Helsinki, Helsinki, Finland; Cornell University

**Keywords:** influenza A, transmission, population bottleneck

## Abstract

The transmission bottleneck is defined as the number of viral particles that transmit from one host to establish an infection in another. Genome sequence data have been used to evaluate the size of the transmission bottleneck between humans infected with the influenza virus; however, the methods used to make these estimates have some limitations. Specifically, viral allele frequencies, which form the basis of many calculations, may not fully capture a process which involves the transmission of entire viral genomes. Here, we set out a novel approach for inferring viral transmission bottlenecks; our method combines an algorithm for haplotype reconstruction with maximum likelihood methods for bottleneck inference. This approach allows for rapid calculation and performs well when applied to data from simulated transmission events; errors in the haplotype reconstruction step did not adversely affect inferences of the population bottleneck. Applied to data from a previous household transmission study of influenza A infection, we confirm the result that the majority of transmission events involve a small number of viruses, albeit with slightly looser bottlenecks being inferred, with between 1 and 13 particles transmitted in the majority of cases. While influenza A transmission involves a tight population bottleneck, the bottleneck is not so tight as to universally prevent the transmission of within-host viral diversity.

**IMPORTANCE** Viral populations undergo a repeated cycle of within-host growth followed by transmission. Viral evolution is affected by each stage of this cycle. The number of viral particles transmitted from one host to another, known as the transmission bottleneck, is an important factor in determining how the evolutionary dynamics of the population play out, restricting the extent to which the evolved diversity of the population can be passed from one host to another. Previous study of viral sequence data has suggested that the transmission bottleneck size for influenza A transmission between human hosts is small. Reevaluating these data using a novel and improved method, we largely confirm this result, albeit that we infer a slightly higher bottleneck size in some cases, of between 1 and 13 virions. While a tight bottleneck operates in human influenza transmission, it is not extreme in nature; some diversity can be meaningfully retained between hosts.

## Abstract (precis)

Viral populations undergo a repeated cycle of within-host growth followed by transmission. Viral evolution is affected by each stage of this cycle. The number of viral particles transmitted from one host to another, known as the transmission bottleneck, is an important factor in determining how the evolutionary dynamics of the population play out, restricting the extent to which the evolved diversity of the population can be passed from one host to another. Previous study of viral sequence data has suggested that the transmission bottleneck size for influenza A transmission between human hosts is small. Reevaluating these data using a novel and improved method, we largely confirm this result, albeit that we infer a slightly higher bottleneck size in some cases, of between 1 and 13 virions. While a tight bottleneck operates in human influenza transmission, it is not extreme in nature; some diversity can be meaningfully retained between hosts.

## INTRODUCTION

Viral populations experience large fluctuations in population size. During the course of an infection, many thousands of viruses may be produced by each infected cell (1), yet in the process of transmission, only a small number of viruses may get through to found a new infection (2). The size of the bottleneck undergone by a viral population at the moment of transmission has an important impact on the evolution of that virus. Where larger numbers of viral particles are involved in transmission, a greater amount of genetic diversity is preserved between hosts; where smaller numbers of particles are transmitted, between-host evolution becomes more of a stochastic process (3). Studying transmission at the scale of individual hosts therefore gives an insight into larger-scale patterns of viral evolution.

Genetic data provide an invaluable insight into processes of viral evolution (4). Such data have been at the core of a variety of approaches for the quantitative analysis of population bottlenecks, typically using observations of minority variants, or their allele frequencies, to make a statistical inference. For example, counting the number of minority variants shared between hosts can be informative of whether transmission occurred between specific hosts (5, 6). If the route of transmission is known, shared variants can be used to estimate the size of the population bottleneck (7). A model of genetic drift may also be applied; smaller or larger changes in the composition of a viral population suggest that a larger or smaller number of viruses was transmitted (3, 8–11). In some situations, engineered viruses with genetic markers have been used to directly evaluate transmission events (12, 13).

Recent studies of influenza transmission between human hosts have used metrics based upon changes in allele frequencies to evaluate the bottleneck at transmission (3, 11, 14, 15). Such metrics have limitations; transmission is ultimately an event in which whole viruses, rather than independent alleles, are passed from one host to another. Neglecting genetic linkage in this way can skew the results of inference methods (16). Based on this finding, a recent study on the assessment of viral transmissibility used sequence data to evaluate transmission at the level of viral genomes (17).

Accounting for genetic linkage between alleles becomes more difficult as the diversity of a viral population increases. In modeling the action of selection on a diverse population, the large number of potential genome sequences can make calculations infeasible. Considering cases in which selection among transmitted variants is not the dominant effect at transmission (3), we here set out an alternative approach for the inference of population bottlenecks, incorporating the true genetic structure of viruses. Our approach has two components. First, given sequence data collected before and after a transmission bottleneck, we apply a method of haplotype reconstruction, using a maximum likelihood framework to calculate a parsimonious reconstruction of the viral population, as observed before and after transmission. A broad variety of computational tools have previously been described for the purpose of haplotype reconstruction in various contexts (18–23); ours fits naturally into the bioinformatic framework we have outlined in previous publications (24, 25). Second, we use the haplotype reconstruction to infer a bottleneck size at transmission; our framework contains two alternative approaches optimized for smaller and larger bottleneck sizes, respectively. We test our method against simulated data describing viral transmission events with a broad range of population bottlenecks. Finally, we reevaluate data from a previous study of influenza transmission between human hosts (3). Our study supports the hypothesis of a generally small transmission bottleneck for influenza viral populations (3, 26), albeit with fractionally higher bottleneck sizes inferred from the same data.

## RESULTS

As a first step, we considered the relative performance of allele- and haplotype-based approaches to the inference of transmission bottlenecks, using grossly simplified, though hopefully illustrative, examples of viral transmission.

### Allele-based versus haplotype-based inference.

**FIG 1:**
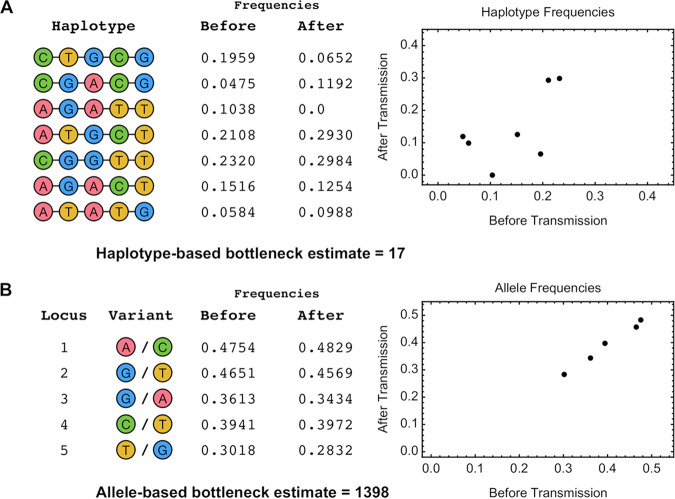
(A) Simulated system of viral transmission. A population comprising seven viral genotypes transmits to a new host, leading to a population in the recipient which includes six of the seven genotypes. A plot shows the sampled frequencies of the distinct genotypes, or haplotypes, before and after transmission, reported to four significant figures. Our explicit model of viral transmission based on haplotype frequencies (described in the text) infers a population bottleneck of 17 viruses from these data. (B) An alternative analysis of the same population measures allele frequencies from the population before and after the transmission event; these are shown in an equivalent plot. A calculation of the population bottleneck from these data infers a value nearly 2 orders of magnitude larger than that of our previous calculation.

A first example highlighted the potential for allele-based statistics to misrepresent the nature of a viral population (Fig. 1). In this simulated system, data were collected from before and after a transmission bottleneck. While during transmission the viral population changed substantially at the genotype level, these changes were not fully reflected in the allele frequency data from each population. As a consequence, inferences of the bottleneck at transmission, calculated using haplotype- and allele-frequency methods, differed by close to 2 orders of magnitude. While an extreme example, this result highlights a fundamental point of biology. Rather than independent alleles, viral transmission involves the transmission of complete viral genomes. Approaches which neglect this may as a consequence be flawed in the results they produce.

**FIG 2:**
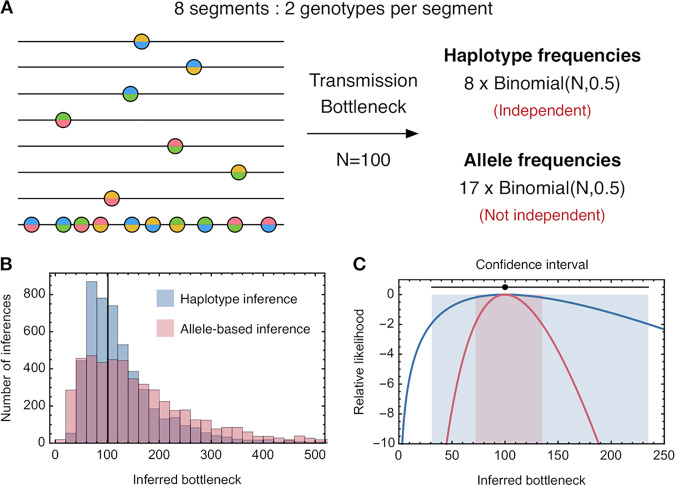
(A) Simulated system of viral transmission. A population consists of eight viral segments. For each segment, two haplotypes exist in the pretransmission population at a frequency of exactly 50%. In seven segments, these haplotypes differ by a single genetic variant, while in the eighth, the haplotypes differ by ten genetic variants. Posttransmission, the haplotype frequencies in each of the eight segments are described by eight independent random binomial samples. The 17 allele frequencies are similarly described by 17 random binomial samples, albeit that these statistics are not independent of each other. (B) Inferred population bottlenecks from 5,000 simulations of this transmission process, calculated with haplotype-based and allele frequency-based methods. A method based upon independent transmission of alleles has an increased variance relative to the haplotype-based method. (C) Likelihood function for each model in the case in which transmission results in a 45/55 split in haplotype frequencies in each segment. The black circle and line indicate the correct transmission bottleneck and an analytical confidence interval based upon a window of two likelihood units. The inference in each case is correct, but the allele-frequency method, which treats the allele frequencies as being statistically independent, has a false level of confidence in the inferred value.

A second example, describing outcomes across a representative range of transmission events, is shown in Fig. 2. We here consider the transmission of a hypothetical influenza viral population. For each segment of the virus, the viral population is divided perfectly into two haplotypes, each with a frequency before transmission of exactly 50%. For seven of the eight viral segments, precisely a single nucleotide polymorphism (SNP) differentiates the 2 haplotypes, while in the final segment 10 SNPs differentiate the haplotypes. In this case, we note that the posttransmission frequency of any given haplotype can be represented as a simple binomial sample from the original population, the chance of any transmitted virus having a certain haplotype being equal to one half. We further note that the same is true for each variant allele; each allele frequency is equal to the frequency of the haplotype which carries it, so that the frequency of the allele is given by a binomial sample. Critically, however, the transmitted haplotype frequencies are independent of one another, while the transmitted allele frequencies are not independent.

The lack of independence has a consequence for the inferred transmission bottlenecks. In the (harmonic) mean, both the haplotype and allele frequency statistics produce a correct inference. However, the allele-based estimate is statistically less precise (Fig. 2B). While in the haplotype inference, each segment is weighted the same, the allele-based estimate is weighted heavily toward the outcome of transmission of the final segment. The variance in the outcome of this one segment is greater than the mean variance across segments, leading the allele-based method to, on average, a worse result. Second, the false assumption in the allele-based method that allele frequencies are independent leads to a false confidence in the outcome of this method (Fig. 2C). The apparently greater amount of data provided by a greater number of polymorphic loci leads to a falsely reduced confidence interval in the bottleneck size at transmission. Where more than one locus is present on a haplotype, and all else being equal, allele frequency methods give less accurate inferences than haplotype-based methods and provide a falsely high level of confidence in their results. We are therefore motivated to consider the transmission of viruses on the genotype level.

To evaluate our genotype-based approach to bottleneck inference, we first considered data describing simulated transmission events, before considering data from a study of human infection.

### Haplotype reconstruction.

**FIG 3:**
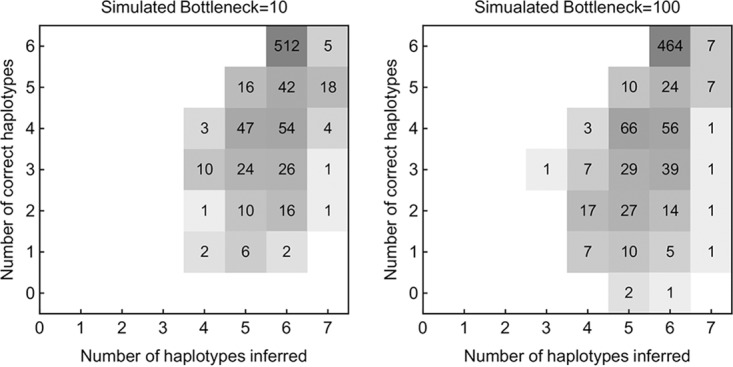
Numbers of inferred and correctly inferred haplotypes given simulated sequence data. A total of 6 haplotypes were included in each of 800 simulations tested.

Applied to simulated data, our method made a correct inference of haplotypes (all existing haplotypes identified, with no false identification of haplotypes) in more than half of the cases tested (Fig. 3). Our approach uses a maximum likelihood method to infer the most parsimonious reconstruction of a viral population, given sequence data. To test our approach, we simulated data describing the transmission of an influenza viral population, from a host to a recipient individual. Each segment in the population was modeled as containing six distinct haplotypes, applying a method for generating data described in a previous study (17). Simulated sequence data from the viral populations in each host were used to infer which haplotypes were present in the transmission event and their frequencies. The most common outcome was a correct reconstruction of all of the haplotypes in the population. We note that our results are particular to the simulation setup; given data from longer genomes, with sparser sequence data, or in a population where haplotypes were present at very low frequency, our method would likely not perform so well by our chosen metric.

### Haplotype-based inference of population bottlenecks.

**FIG 4:**
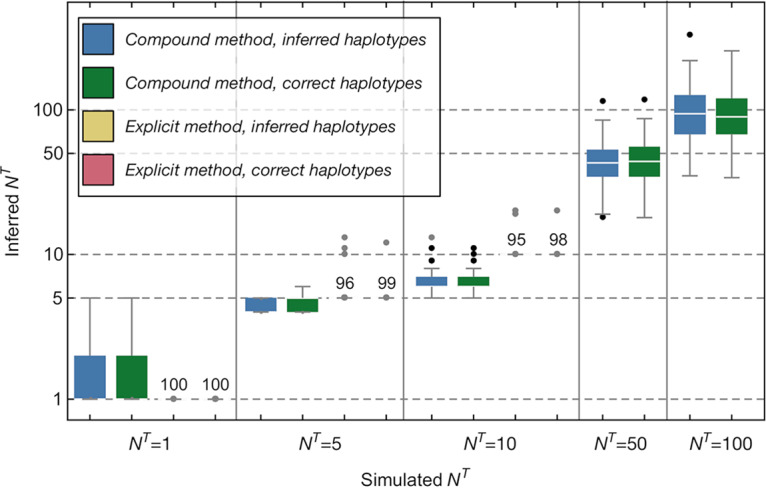
Transmission bottleneck sizes inferred from simulated data using different input data and methodologies. Inferences are shown in color according to the data and method used. Calculations with inferred haplotypes took as input data generated from a haplotype reconstruction method applied to simulated sequence data in which both the haplotypes and their frequencies before and after transmission were inferred. Calculations with the correct haplotypes took as input data from a haplotype reconstruction in which the identities of the correct haplotypes were given, with only their frequencies being inferred. Inferences from the explicit method were only calculated for smaller population bottleneck sizes, as the method does not scale well to evaluating larger bottlenecks. Results from the explicit method were so accurate as to not have a meaningful interquartile range; numbers displayed in these cases indicate the number of inferences giving a precisely correct inference of the population bottleneck. Horizontal dashed lines indicate the simulated bottleneck sizes.

Our two methods for bottleneck inference produced good results when applied to simulated viral transmission data (Fig. 4). As described in Materials and Methods, our two methods generalize the approaches of two previously described single-locus methods for bottleneck inference (11, 14). Our “compound method” uses a model of genetic drift in a continuous space of genotype frequencies, in which smaller changes in frequencies correspond to a lesser amount of stochasticity in transmission and hence a larger population bottleneck (14). Our “explicit method” explicitly evaluates all of the possible outcomes of a transmission event across a discrete space; the fact that an integer number of viruses of each genotype are transmitted is used to weigh up the likelihood of different potential bottlenecks (11).

When we applied these methods to simulated data, the compound method generally did well, inferring transmission bottlenecks that were close to the simulated values. One advantage of this method is that its running time does not increase with the bottleneck size, enabling the analysis of very high potential bottleneck sizes. A disadvantage of the method is that, despite improvements made with respect to its predecessor (17), the mathematical approximations made in its construction mean that it does not always perform so well at low bottleneck sizes, producing a visible underestimate of bottlenecks of size 10.

Further inferences of bottleneck size were made using reconstructions of haplotypes in which the correct simulated haplotypes were prespecified, learning only their frequencies. Using these improved data did not produce a noticeable improvement in the inference of the bottleneck size, suggesting that our inference of bottleneck size is robust to errors that arise from our haplotype reconstruction method. Bottleneck sizes in each case were calculated across eight independent viral segments.

Given our simulated data, the explicit method outperformed the compound method at low bottleneck sizes, inferring exactly correct values in the majority of cases with very little error. A disadvantage of the explicit method is that in requiring the evaluation of all possible outcomes of a transmission event, the computational time it requires grows very rapidly as the bottleneck size increases. For this reason, we did not apply it to data from higher simulated population bottlenecks. As with the compound method, performance did not greatly improve given frequencies inferred using the correct viral haplotypes; errors in haplotype reconstruction did not have a strong effect on the inferred bottleneck sizes.

The variances in the inferred bottleneck sizes are dependent upon the amount of data available to our code for inference. In the case of a less diverse viral population, less genetic information would be available, leading to a greater variance in the inferred bottlenecks. By contrast, more diversity would lead to a more constrained inference. Data shown here are intended to illustrate the mean performance of our methods.

Inference of bottleneck size for a segment was not possible in two cases. First, if our haplotype reconstruction found evidence for only a single viral haplotype, no inference was possible, insufficient information about the event being available. Second, if the viral population in the recipient was inferred to have arisen purely from a *de novo* haplotype, which had swept to fixation in the population between the establishment of the infection and the collection of the sequence data, this result was uninformative in identifying a bottleneck. In either of these circumstances, data from a viral segment were ignored, inferences conducted for the remaining segments being combined to infer the final bottleneck size.

In considering the differences in inferences achieved by the two methods at low bottleneck sizes, it is perhaps helpful to consider the simple case where a single allele frequency is observed to change from 50% frequency in the donor to 5% in the recipient. Within the compound method, this represents a large change in allele frequency, corresponding to a large amount of genetic drift, and will be interpreted as resulting from a low bottleneck size. In contrast, under the explicit method, variation at a frequency of 5% is unlikely to be observed if the bottleneck is low; at least one particle with the variant must have been transmitted, implying a minimum variant frequency of at least 1/*N^T^*. Transmission with a bottleneck closer to 20, with sampling noise leading to the underestimation of the variant frequency, would give a more coherent explanation.

### Application to data from a household study.

Our transmission model was applied to data collected from a previously published household study (3). This study used a single-locus inference model to identify narrow bottlenecks in human-to-human transmission, with all but a single event being inferred to involve the transmission of between one and four viral particles. Short-read data from this study were filtered and processed into variant data before being fed into our method. Having identified polymorphic loci in pairs of transmission data using an allele frequency cutoff of 2%, we generated multilocus reads from the data using the SAMFIRE software package (25), using these to generate an inference of haplotype frequencies before and after transmission. These frequencies were used to infer population bottleneck sizes for each transmission event.

**FIG 5:**
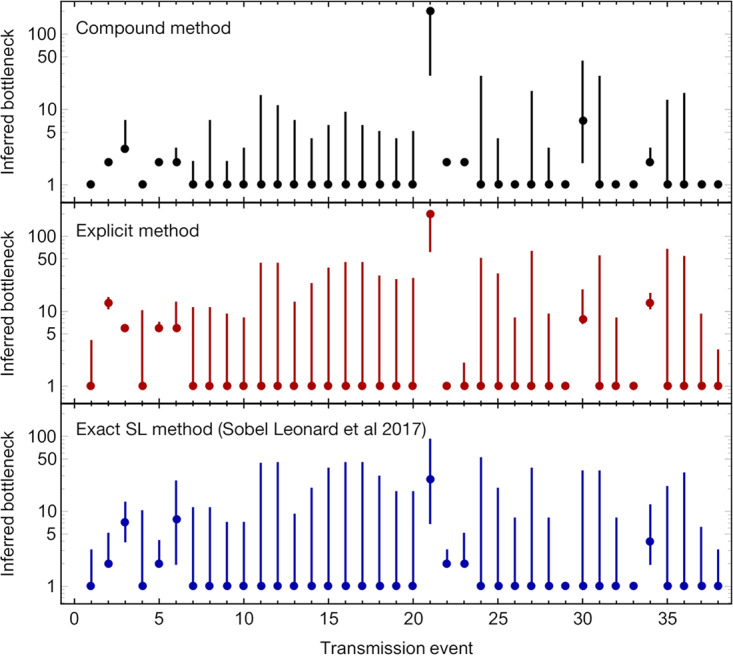
Bottleneck sizes inferred from the data presented in reference 3. Dots indicate the maximum likelihood bottleneck size inferred for each of the 38 systems in this work for which we were able to infer a bottleneck. Vertical bars represent confidence intervals of 2 log likelihood units from the maximum.

We confirm the previous inference of tight population bottlenecks in all cases (Fig. 5). In the majority of transmission events (29 out of 38 events for which we obtained an inference), bottlenecks of size *N^T^* = 1 were inferred by both of our methods, consistent with all of the diversity of the viral population in the original host being lost at transmission. While not necessarily implying that these infections were started by a single viral particle, these results are consistent with the hypothesis of a generally tight bottleneck at transmission. In 8 out of the remaining 9 transmission events, intermediate bottleneck sizes were inferred, with a range from 2 to 7 in the compound method and from 2 to 13 in the explicit method. Evidence from simulated data suggests that the explicit method is probably more accurate in this range. Finally, there was a single case in which a bottleneck size of 200 or more was inferred; this was set as the upper limit considered by our study. Our inference in this case matched the original analysis of the data. A further statistical analysis of the samples collected before and after transmission indicated a greater degree of similarity between allele frequencies than was previously found in a case where replicate clinical samples were processed and sequenced in parallel (27). Whereas in the previous study, measurements of allele frequencies from samples split from the cDNA synthesis step onward were consistent with an effective read depth (which is equivalent to an error-free sample depth) of 1,000 or more, here an effective depth in excess of 20,000 was inferred, demonstrating that the before- and after-transmission samples were extremely similar. This case could represent either a very unusual transmission event, in which an extreme number of viruses were transmitted, or potentially an isolated error in the processing of a large number of sequence samples.

Cases in which the explicit method inferred larger bottleneck sizes than the compound method could be explained in terms of the preservation of allele frequencies at relatively low frequencies; as explained above, the explicit method can favor a higher bottleneck in such cases.

Our approach was not able to infer a population bottleneck in five of the transmission cases analyzed by the original study. In these cases, a low level of polymorphism observed before transmission was no longer present after transmission. Application of our haplotype reconstruction method in these cases did not find statistical evidence for more than one haplotype (plus noise) in these systems, at least two specific haplotypes being required for an inference of bottleneck size. We understand this in terms of our haplotype reconstruction method being less sensitive to detecting variation than is the 2% allele frequency cutoff used in the original study; the presence of a variant allele at 2% frequency was not always sufficient evidence for our code to infer the existence of two specific genetic variants in the population. In these cases, the loss of host genetic variance at transmission would lead our methods to the conclusion that a bottleneck of *N^T^* = 1 best explained the observed data, strengthening our main result of a tight bottleneck size. The sensitivity of our method in calling additional haplotypes can be somewhat arbitrarily tuned.

Differences in the bioinformatic processing of data could underlie some of the differences in bottlenecks we identified. While we replicated the 2% allele frequency cutoff of the original paper (3), we called variants in 18 of the 38 transmission events analyzed here that were not originally found. Such variants were primarily only found in one of the two samples and existed at frequencies very marginally above the 2% threshold; minor allele frequencies very close to the threshold were observed both in our processing of the data and in the original study (see Table S1 in the supplemental material). Applying the exact single-locus method for bottleneck inference of a previous study (for convenience, we term this the exact SL method) (11) we found cases of higher bottlenecks than were found in the original paper (Fig. 5). In common with the original study, we remove variants in noncoding regions of the genome from our calculation.

Bioinformatic variations in the calling of alleles can have three distinct effects. Where an additional variant is called in the recipient population but not in the donor, no change in the inferred bottleneck occurs; the variant is assumed to have arisen *de novo* in the recipient, having nothing to do with the transmission event. Where an additional variant is called in the donor population but not in the recipient, this shifts the inference toward a smaller bottleneck. The dying out of a low-frequency variant is the most likely outcome given a small bottleneck, so this usually makes little difference to the inference. However, in transmission event 21, we observe that a bottleneck inferred to involve at least 200 particles by both of our haplotype-based methods (and the original study) was inferred to involve only 29 particles by the exact SL method. In this case our bioinformatic approach called two variant alleles, NA G1351A and PB1 A2280G, at 2.3% and 3.2% in the donor population, which died out upon transmission. Our haplotype inference method did not find sufficient evidence to identify two haplotypes for these segments and ignored these variants as a result, but the exact SL method accounted for them, leading to a reduced bottleneck inference. Especially at high bottlenecks, small bioinformatic changes can have an important effect.

Finally, where an additional variant is called in both the donor and the recipient populations, it can influence the inferred bottleneck in either direction. Four such cases were found in our analysis, in transmissions 2, 3, 5, and 6. Removing these variants from the populations led to a reduction in the bottleneck inferred under the explicit method to a single particle for transmissions 2, 3, and 6. The inferred bottleneck for transmission 5 was slightly reduced from *N^T^* = 6 to *N^T^* = 5. Not all of the cases in which bottlenecks of greater than 1 were inferred could be explained by bioinformatic variation. The inference of *N^T^* = 13 in transmission 34 had a single additional variant in our processing that was not found in the original analysis, consisting of a low-frequency variant that was not transmitted to the recipient host. As noted above, such a variant could not increase the size of the inferred bottleneck.

## DISCUSSION

We have here set out a haplotype-based approach for the inference of transmission bottlenecks and demonstrated its application using data from a study of transmission of influenza A infection.

Haplotype-based methods have the advantage of faithfully representing the biological event of viral transmission. While the use of allele frequency statistics does not necessarily lead to incorrect results, such use introduces a level of abstraction from reality. In some cases, this can lead to grossly misleading results; in general, it will give a less precise inference of bottleneck size and a falsely high level of confidence in the results obtained. The shortfall in performance of an allele-based method will depend upon the system in question. In a hypothetical influenza virus with only a single variant per segment, allele- and haplotype-based approaches will likely give identical results. In a nonsegmented virus, with high viral diversity, the assumption of independent alleles will lead to a substantial overestimation of the statistical confidence with which a bottleneck can be quantified.

We used a haplotype reconstruction method to infer the composition of the viral population before and after transmission; by requiring substantial evidence to add an additional haplotype to the model, this approach limits the complexity of the inferred viral population, improving the feasibility of haplotype-based bottleneck inference relative to a previous approach (17). While our haplotype reconstruction method was not perfect in reproducing the details of a viral population, errors resulting from this method did not greatly harm our inference of population bottleneck sizes.

Our approach for bottleneck inference comprises two distinct methods, designed for use at high and low bottleneck sizes. The first of these generalizes the approach of Poon et al. (14), who used a formula based on genetic drift to evaluate changes in allele frequencies. Our compound method generalizes this to changes in haplotype frequencies, which occur in higher-dimensional sequence space; it further incorporates uncertainty in the inferred haplotype frequencies and genetic drift arising from within-host population growth. This method has the advantage of being rapid to calculate at high bottleneck sizes but potentially underestimates bottleneck sizes at low values of *N^T^*. Our second method, the explicit method, generalizes the approach of Sobel Leonard et al. (11), who apply a beta-binomial formula to evaluate possible discrete outcomes of a transmission process. In spirit, we repeat this approach, summing a likelihood function over the set of possible outcomes of a transmission of viral haplotypes. This approach is limited in its application to systems of higher complexity, becoming slow where there are many haplotypes or where *N^T^* is large, but is likely more accurate at lower bottleneck sizes. The size of a bottleneck affects the two methods in different ways. For the compound method, increased bottleneck size leads to greater accuracy, in that the mathematical approximations underlying the method become increasingly correct as the product between the bottleneck size and a typical haplotype frequency increases. For the explicit method, increased size adversely affects the time required for calculation, in that as the number of haplotypes in the system and the bottleneck size become large, the evaluation over all possible outcomes of a transmission event becomes increasingly intractable.

While our haplotype reconstruction and bottleneck inference methods are constructed upon a common likelihood framework, our inference methods could be applied to haplotype data from other sources. Other reconstruction methods could provide appropriate data for analysis, while barcoding technologies or long-read sequencing could each obviate the need for a reconstruction step. We note that, where ethically feasible, the use of neutral markers provides a more direct approach for evaluating transmission events (12).

Our framework makes the assumption of selective neutrality during the transmission event. Selection during transmission, whether positive or negative, changes the genetic composition of the viral population in the recipient relative to that of the donor. On average, this makes the population in the recipient less similar to that in the donor, leading to an underestimate of the population bottleneck. A variant of our compound method incorporating selection has been set out in a previous publication (17). Evaluating selection requires a comprehensive reconstruction of the extant viral haplotypes; this may be difficult to obtain given short-read data describing a diverse population. Identifying variants that enhance viral transmissibility is impossible where very few viruses are transmitted; at higher population bottlenecks or where multiple transmissions are observed, it becomes an achievable task. Under selection, haplotype-based approaches have further advantages over allele-frequency statistics (16).

As we have shown, apparently small differences in the calling of variants can have significant consequences for the inference of bottleneck sizes. Regardless of the method used for inference, if a variant was falsely called to exist at low frequency in both the pre- and posttransmission populations, this could dramatically skew an inference toward a higher bottleneck size. Our reanalysis of data preserved the frequency cutoff for alleles used by the original authors but nevertheless found additional variants in excess of this cutoff, likely the result of fractional changes in the bioinformatic processing. Marginal frequencies close to the frequency cutoff were identified both in our processing of the data and in the output of the original study. Where a hard cutoff is used for variant identification, and specific variants are close to this cutoff, uncertainty in the identification or nonidentification of variants should be considered part of the uncertainty in bottleneck inference; statistical approaches for this could provide an area for future development.

Progress in understanding the biology of infection could be a further aid in the development of methods for bottleneck inference. In particular, the dynamics of the very early stages of population growth, from the initial founder viruses to the large population typical of influenza infection, are not necessarily well understood. Knowledge of the extent to which this affects the genetic composition of the viral population would improve the potential for accurate inference.

We have here used a haplotype-based approach to study transmission bottlenecks using data from a household study of influenza A infection. While we replicate the finding that transmission involves a small number of viral particles, our results have a longer tail of bottleneck sizes, with estimates of up to 13 viruses being transmitted. While transmission may strongly limit the inheritance of influenza virus diversity, its effect in doing so is not absolute; the transmission of viral diversity may occur and have some influence on broader viral evolutionary dynamics.

## MATERIALS AND METHODS

### Notation.

**FIG 6:**
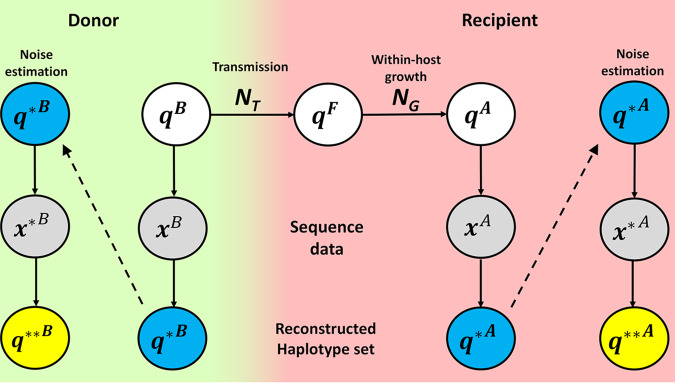
Notation in the transmission model. Transmission of the population ***q****^B^* with bottleneck *N^T^* results in the founder population ***q****^F^*. The founder population grows under the influence of genetic drift, the effects of which are described by the effective population size *N^G^*. Growth results in the population ***q****^A^*. The populations ***q****^B^* and ***q****^A^* are observed, producing data sets represented by ***x****^B^* and ***x****^A^*, which are used to reconstruct the original populations in terms of haplotypes. In order to calculate the variance of the reconstructed populations q*^B^ and q*^A^, data sets equivalent to ***x****^B^* and ***x****^A^*, denoted x*^B^ and x*^A^, are generated and used to infer sets q**^B^ and q**^A^.

A guide to the notation used in our methods is shown in Fig. 6. Briefly, we represent the populations before and after transmission by vectors of unknown haplotype frequencies, referred to as ***q****^B^* and ***q****^A^*, respectively. These are separated by transmission with a bottleneck, *N^T^*, forming the founder viral population ***q****^F^* in the recipient and then within-host growth, represented in our model by a single generation of genetic drift with effective size *N^G^*. The unknown vectors ***q****^B^* and ***q****^A^* are indirectly observed via the data sets ***x****^B^* and ***x****^A^*, which are used to generate the estimated haplotype frequencies q*^B^ and q*^A^.

In generating the variance of our estimates, we use q*^B^ and q*^A^ to generate simulated observations, which we term x*^B^ and x*^A^. These in turn are used to generate a new round of estimates, q**^B^ and q**^A^. In so far as q**^B^, q**^A^, q*^B^, and q*^A^ are all known, they may be used to estimate the variances of q*^B^ and q*^A^.

### Haplotype reconstruction.

We developed a maximum likelihood approach for haplotype reconstruction based upon existing technologies for processing short-read data (24, 25, 27). We assume here that we have short-read data describing a viral population both before and after a transmission event. Before commencing haplotype reconstruction, we performed three steps to preprocess the data using our software package SAMFIRE (25). First, after alignment to the viral genome using the Burrows-Wheeler Aligner (BWA) (28), the short-read data were filtered, trimming reads to achieve a median Phred score of at least 30, combining data from paired-end reads, and removing individual base calls with a Phred score of less than 30. Second, the filtered data were used to identify loci at which a polymorphism existed at significant frequency, this being defined using a cutoff of 2% to match the study of McCrone et al. (3), from which we obtained the data we analyzed. Third, reads were processed to generate partial haplotypes, which describe the nucleotides present at each of the polymorphic loci in each read. Partial haplotype data were divided into distinct sets of reads, each describing alleles at a distinct set of loci in the viral genome. As an optional step, an estimate may be produced of the extent of noise present in sequence data, inferring a parameter, *C*, which describes the precision with which measurements of allele frequencies may be calculated via sequencing (25). A value of *C *= 1 here corresponds to a case in which reads are uninformative, while large values of *C* tend toward the binomial case in which each read accurately describes the allele present in a distinct viral genome, sampled in an unbiased manner from the population. A default value of *C *= 200 was used for our simulations.

We denote the sets of partial haplotype data collected before and after transmission as $x_{l}^{B,P}$ and $x_{l}^{A,P}$, respectively, where *l* denotes the partial haplotype set. We now suppose that the viral population comprises a set of distinct haplotypes, denoted ***H***, which comprises *k* haplotypes, having the frequencies $q^{B}=\left\{q_{i}^{B}\right\}$ before transmission and $q^{A}=\left\{q_{i}^{A}\right\}$ after transmission. These frequencies can be converted into partial haplotype frequencies by projection of the full haplotype space onto each lower-dimensional partial haplotype space by means of matrices *T_I_*. For example, given the full haplotypes before transmission {GA, TA, GC, TC} and a set of partial haplotypes {G-, T-}, we may write

(1) $$q_{l}^{B,P}=T_{l}q^{B}$$

or more explicitly,

(2) $$\left(\begin{array}{c} q_{l,1}^{B,P}\\ q_{l,2}^{B,P} \end{array}\right)=\left(\begin{array}{llll} 1 & 0 & 1 & 0\\ 0 & 1 & 0 & 1 \end{array}\right)\left(\begin{array}{l} q_{1}^{B}\\ q_{2}^{B}\\ q_{3}^{B}\\ q_{4}^{B} \end{array}\right)$$

In the above instance, we note that each partial haplotype can potentially be emitted from at least one of the haplotypes in H. In order to generalize our model, we included in each set H a further haplotype “X,” describing the cloud of all potential viral haplotypes of the same length as those in H yet not already defined as being in H. With this inclusion, we may say that any potential partial haplotype may be emitted from at least one of the haplotypes in H, being emitted either from one of the defined haplotypes or from “X.”

In this way, we can construct a likelihood for any given set of haplotypes and frequencies, given the partial haplotype data. We write

(3) $$\text{log }L(H)=\underset{t\in \{B,A\}}{\sum }\underset{l}{\sum }\text{log }L_{D}\left(x_{l}^{t,P}|T_{l}q^{t},C\right)$$

where $L_{D}$ denotes the Dirichlet multinomial likelihood

(4) $$L_{D}(x|q,C)=\frac{\Gamma (N+1)}{\prod_{i}\Gamma (x_{i}+1)}\frac{\Gamma \left(\underset{i}{\sum }Cq_{i}\right)}{\Gamma \left(N+\underset{i}{\sum }Cq_{i}\right)}\prod_{i}\frac{\Gamma \left(x_{i}+Cq_{i}\right)}{\Gamma \left(Cq_{i}\right)}$$

in which *N* = Σ_i_ *x_i_*.

A two-step optimization was used to infer the optimal set of haplotypes and frequencies. To construct an initial set H, a set of *k *≥ 1 unique haplotypes were created in turn, to which was added the additional X haplotype. The frequencies of these haplotypes before and after transmission were then optimized under the constraint that the frequency of the X haplotype could not be greater than 0.01; this prevents the inference of trivial solutions to the model. These inferred haplotype frequencies specify q*^B^ and q*^A^. We note that the frequency of the X haplotype may be effectively zero; for the purposes of calculation, a minimum frequency of *ε* = 10^−20^ was imposed.

Given our likelihood function, a series of changes were made to the set ***H***, optimizing the frequencies each time to find the optimal haplotype reconstruction. Repeating this for increasing values of *k* gives a series of fits to the data; we used the Bayesian information criterion (BIC) to distinguish the most parsimonious explanation for the data:

(5) $${\text{BIC}}_{k}=-2L^{*}\left(H_{k}^{*}\right)+k\text{ log }N$$

where $L^{*}\left(H_{k}^{*}\right)$ is the optimum likelihood value for the optimal set $H_{k}^{*}$ of *k* haplotypes, and *N* is the total number of observations in the data set. Optimization of the haplotype set was conducted for increasing values of *k* until a model with an additional haplotype produced an improvement of less than 10 units of BIC, representing a conservative cutoff point; a smaller required improvement would lead to the inference of a greater number of haplotypes. In our model, the same *k* haplotypes had to be used for the reconstructions of both the pre- and posttransmission samples. Our model retained the possibility of haplotypes having zero frequency after transmission, for example, in the case of a tight bottleneck, or before transmission, in the case of the emergence of a *de novo* mutation following a transmission event.

### Estimated error in reconstructed haplotype frequencies.

For our compound method for bottleneck inference, we require an estimate of the variance in the inferred haplotype frequencies q*^B^ and q*^A^, so as to account for noise in these parameters when evaluating changes in the population. Variances were calculated by means of simulated data. Considering data collected before transmission, we used the frequencies q*^B^ to generate sets of partial haplotype data $x_{l,j}^{*B,P}$, where *j* is used to index different sets. Each set provided an independent statistical replicate of the original data, having an identical number of sets of partial haplotypes, each spanning the same loci and containing the total number of samples. Each set was generated using a random Dirichlet multinomial sampling process with value *C* identical to the original. For each set of data, the haplotype reconstruction process was repeated, but with the haplotypes ***H*** constrained to those inferred for the original data. This process was repeated for 100 sets of data, generating the inferred haplotype frequencies $\left\{q_{j}^{**B}\right\}$. These values were used to calculate the diagonal elements of a covariance matrix var[q*^B^] for q*^B^, given by

(6) $$\text{var}{\left[q^{*B}\right]}_{i,i}=\frac{1}{100}\sum_{j=1}^{100}{\left(q_{i}^{*B}-\left\{q_{\mathrm{ij}}^{**B}\right\}\right)}^{2}$$

For simplicity, off-diagonal elements of this matrix were set to zero. An identical process was used to generate the matrix var[q*^A^].

### Allele-frequency models of bottleneck inference.

In generating Fig. 1 and 2 we used a simple single-locus model of bottleneck inference. Given a set of independent allele frequencies $q_{i}^{B}$ at locus *i* in the pretransmission viral population, and their equivalent values $q_{i}^{A}$ in the posttransmission population, we note that in the absence of selection, the mean value of $q_{i}^{A}$ is given by $q_{i}^{B}$, while the variance of $q_{i}^{A}$, arising from genetic drift in a haploid system, is given by

(7) $$V=\frac{q_{i}^{B}\left(1-q_{i}^{B}\right)}{N}$$

where *N* is the effective population size of the system (29).

To estimate the bottleneck size at transmission, we made the approximation that $q_{i}^{A}$ is normally distributed and then maximized the sum of the log likelihood values across allele frequencies

(8) $$L\left(N^{T}\right)=\sum_{i}\text{log }N\left(q_{i}^{B},\frac{q_{i}^{B}\left(1-q_{i}^{B}\right)}{N^{T}}\right)$$

where *N^T^* is the transmission bottleneck and the sum is calculated over loci *i* with polymorphic alleles. In the case where only two haplotypes are observed in a segment, this approach can be applied to haplotype, rather than allele, frequencies. This was done for the haplotype-based calculations in Fig. 2.

In the analysis of influenza sequence data, we applied the exact version of the beta-binomial sampling method described by Sobel Leonard et al. (11). This method identifies the value of *N^T^* that maximizes the likelihood

(9) $$L\left(N^{T}\right)=\sum_{i}\sum_{k=0}^{N^{T}}\left(\begin{array}{l} x_{i}^{A}\\ n_{i}^{A} \end{array}\right)\frac{B\left(n_{i}^{A}+k,x_{i}^{A}-n_{i}^{A}+N^{T}-k\right)}{B\left(k,N^{T}-k\right)}\left(\begin{array}{c} N^{T}\\ k \end{array}\right){\left(q_{i}^{B}\right)}^{k}{\left(1-q_{i}^{B}\right)}^{N^{T}-k}$$

where $x_{i}^{A}$ is the total number of reads at locus *i*, $n_{i}^{A}$ is the number of reads at *i* which describe the variant allele, *B*(*α*,*β*) is the beta function, and the outer sum is conducted over polymorphic loci.

### Haplotype-based methods of bottleneck inference.

Frequencies inferred from the haplotype reconstruction were used for the explicit and compound methods for calculating bottleneck size. As a first step in each method, we removed haplotypes that were inferred to have been created *de novo* in the recipient following the transmission by removing haplotypes for which the pretransmission frequency fell below a threshold frequency δ, set by default to 0.5%. Elements of the vectors q*^B^ and q*^A^ and the respective rows and columns of their covariance matrices were removed in this preliminary step.

In so far as we consider influenza transmission, we consider data from each viral segment independently, calculating first a likelihood of the bottleneck size given data from each segment before combining the likelihoods across segments to estimate an overall maximum likelihood value for the transmission bottleneck.

### Compound method for bottleneck estimation.

In the case of larger values of *N^T^*, an approach building upon that described in a previous publication (17) was applied. Briefly, we note that in a neutral transmission bottleneck, the expected composition of the population in the recipient is identical to that in the original host. The variance in this population is then a function of the size of the bottleneck and the extent of genetic drift during within-host growth, while in the case of inference, variation arising from the measurement of each population must also be considered.

Similar to the approach outlined in an earlier work (17), we calculate a likelihood function with two components:

(10) $$\begin{array}{l} L\left(N^{T}|q^{*B},q^{*A},N^{G}\right)=\int P\left(q^{*B}|q^{B}\right)P\left(q^{B}\right)dq^{B}\\ \times \int P\left(q^{*A}|q^{A}\right)\{\int P\left(q^{A}|N^{G},q^{F}\right)\\ \times \left(\int P\left(q^{F}|N^{T},q^{B}\right)P\left(q^{B}\right)dq^{B}\right)dq^{F}\}dq^{A} \end{array}$$

where the first integral corresponds to the initial observation of the system and the second encompasses transmission (with the bottleneck *N^T^*), within-host growth (with drift described by the effective size *N^G^*), and posttransmission sampling. Each component of the likelihood is relatively simple to consider, as either a multinomial or Dirichlet-multinomial process, but the compound is difficult to evaluate. We note that, in cases where the frequency of a haplotype remains far from 0 or 1, and in particular, as *N^T^* becomes large, the likelihood can be increasingly well approximated in terms of a Gaussian distribution, with mean and variance calculated below.

Our solution makes use of the laws of total expectation and total variance. Given distributions *U* in *x* and *V* in *y*, the compound distribution *W* takes the form

(11) $$P_{W}(x)=\int P_{U}(x|y)P_{V}(y)\text{d}y$$

The mean and variance of *W* are then defined by

(12) $${\text{E}}_{W}[x]={\text{E}}_{V}\left[{\text{E}}_{U}[x|y]\right]$$

and

(13) $${\text{var}}_{W}[x]={\text{E}}_{V}\left[{\text{var}}_{U}[x|y]\right]+{\text{var}}_{V}\left[{\text{E}}_{U}[x|y]\right]$$

respectively.

For the pretransmission component, the calculation of mean and variance are simple; our haplotype reconstruction process gives the estimate

(14) $$\text{E}\left[q^{B}\right]\approx q^{*B}$$

where the right-hand side is the output of the haplotype reconstruction, and

(15) $$\text{var}\left[q^{B}\right]\approx \text{var}\left[q^{*B}\right]$$

where the right-hand side was calculated using the generation of the data sets $x_{l,j}^{*B,P}$ and the inferences of the frequencies {q**^B^}*_j_*.

Moving on to the posttransmission component of the compound distribution in equation 10, we can carry out the relevant marginalizations using the law of total expectation and the law of total variance.

Given that the dynamics governing transmission and within-host growth are assumed to be selectively neutral, the mean frequencies of the viral population are unchanged following transmission and growth. The mean term is therefore straightforward to calculate.

(16) $$\begin{array}{l} \text{E}\left[q^{*A}\right]=\text{E}\left[\text{E}\left[q^{*A}|q^{A}\right]\right]=\text{E}\left[q^{A}\right]\\ \text{E}\left[q^{A}\right]=\text{E}\left[\text{E}\left[q^{A}|q^{F}\right]\right]=\text{E}\left[q^{F}\right]\\ \text{E}\left[q^{F}\right]=\text{E}\left[\text{E}\left[q^{F}|q^{B}\right]\right]=\text{E}\left[q^{B}\right] \end{array}$$

Thus,

(17) $$\text{E}\left[q^{*A}\right]\approx q^{*B}$$

Calculation of the variance requires a little more effort. The transmission event can be modeled as a single multinomial draw with *N^T^* number of trials. As a result, the variance of the founder population is given by

(18) $$\text{var }\left[q^{F}|q^{B}\right]=\frac{1}{N^{T}}M\left(q^{B}\right)$$

where *M*(***q***) = Diag(***q***) – ***qq***.

We therefore obtain that

(19) $$\begin{array}{l} \mathrm{var}[q^{F}]=\text{E}[\mathrm{var}\left[q^{F}{|q}^{B}\right]]+\mathrm{var}\left[\text{E}\left[q^{F}{|q}^{B}\right]\right]\\ =\text{ E}[\frac{1}{N^{T}}M\left(q^{B}\right)]+\mathrm{var}\left[q^{B}\right]\\ =\frac{1}{N^{T}}(\text{E}[\text{Diag}\left(q^{B}\right)]-\text{E}\left[q^{B}{\left(q^{B}\right)}^{†}\right])+\mathrm{var}\left[q^{B}\right]\\ =\frac{1}{N^{G}}(\text{Diag}(\text{E}\left[q^{B}\right])-\mathrm{var}[q^{B}]-\text{E}[q^{B}]E{\left[q^{B}\right]}^{†})+\mathrm{var}\left[q^{B}\right]\\ =\frac{1}{N^{T}}M(\text{E}\left[q^{B}\right])+(1-\frac{1}{N^{T}})\mathrm{var}\left[q^{B}\right]\\ \approx \frac{1}{N^{T}}M(q^{B})+(1-\frac{1}{N^{T}})\mathrm{var}\left[q^{*B}\right] \end{array}$$

where we used the result

(20) $$\text{E}\left[qq^{†}\right]=\text{var}[q]+\text{E}[q]\text{E}{\left[q\right]}^{†}$$

The within-host growth dynamics can be modeled as a multinomial draw of depth *N^G^* = *gN^T^*, where *g* is the growth factor. From this we obtain the result that

(21) $$\text{var}\left[q^{A}|q^{F}\right]=\frac{1}{N^{G}}M\left(q^{F}\right)$$

Marginalizing over ***q****^F^*, we obtain the variance

(22) $$\begin{array}{l} \mathrm{var}[q^{A}]=\text{E}[\mathrm{var}\left[q^{A}|q^{F}\right]]+\mathrm{var}\left[\text{E}\left[q^{A}|q^{F}\right]\right]\\ =\text{ E}[\frac{1}{N^{G}}M\left(q^{F}\right)]+\mathrm{var}\left[q^{F}\right]\\ =\frac{1}{N^{G}}(\text{E}[\text{Diag}\left(q^{F}\right)]-\text{E}\left[q^{F}{\left(q^{F}\right)}^{†}\right])+\mathrm{var}\left[q^{F}\right]\\ =\frac{1}{N^{G}}(\text{Diag}(\text{E}\left[q^{F}\right])-\mathrm{var}[q^{F}]-\text{E}[q^{F}]E{\left[q^{F}\right]}^{†})+\mathrm{var}\left[q^{F}\right]\\ =\frac{1}{N^{G}}M(\text{E}\left[q^{F}\right])+(1-\frac{1}{N^{G}})\mathrm{var}\left[q^{F}\right]\\ \approx \frac{1}{N^{G}}M(q^{*B})+(1-\frac{1}{N^{G}})\left(\frac{1}{N^{T}}M(q^{*B})+(1-\frac{1}{N^{T}})\mathrm{var}\left[q^{*B}\right]\right)\\ =\frac{N^{T}+N^{G}-1}{N^{T}N^{G}}M(q^{*B})+\frac{N^{T}N^{G}-N^{T}-N^{T}+1}{N^{T}N^{G}}\mathrm{var}\left[q^{*B}\right]\\ \equiv \gamma M[q^{*B}]+\delta \,\mathrm{var}[q^{*B}], \end{array}$$

where we define $\gamma =\left(\frac{N^{T}+N^{G}-1}{N^{T}N^{G}}\right)$ and $\delta =\frac{N^{T}N^{G}-N^{T}-N^{G}+1}{N^{T}N^{G}}$

Finally, we have that

(23) $$\begin{array}{c} \text{var}\left[q^{*A}\right]=\text{E}\left[\text{var}\left[q^{*A}|q^{A}\right]\right]+\text{var}\left[\text{E}\left[q^{*A}|q^{A}\right]\right]=\text{E}\left[\text{var}\left[q^{A}\right]\right]+\text{var }\left[q^{A}\right]\\ =\text{ var}\left[q^{*A}\right]+\gamma M\left(q^{*B}\right)+\delta \text{var}\left[q^{*B}\right]. \end{array}$$

Together, equations 16 and 23 define the mean and variance of a multivariate normal distribution representing the posttransmission component of the likelihood in equation 10. Given our inferences for q*^B^ and q*^A^, we optimized the likelihood with respect to *N^T^*, generating a maximum likelihood estimate for the bottleneck size. We note that our approximation of the likelihood in terms of a multivariate normal distribution works best where individual haplotype frequencies are not too close to zero or one, and where *N^T^* is large. However, the approach allows for rapid calculation. In this sense we say that the compound method is optimized for large *N^T^*.

### Correction for the extinction of haplotypes in the compound method.

Where a haplotype goes extinct in the transmission process, the likelihood function of the compound method can provide a poor estimate to the correct value. In this special case, relevant in our simulated data, we used a conditional distribution approach to make a correction to the likelihood.

In the above approximation we generated a multivariate normal distribution for q*^A^:

(24) $$q^{*A}∼N\left(q^{*B},\text{var}\left[q^{*A}\right]\right)$$

In this context, we split the vector q*^A^ into $q_{1}^{*A}$ and $q_{2}^{*A}$, the latter containing all haplotypes posttransmission with a frequency lower than the threshold frequency η, which were considered to have died out during transmission, and the former containing the “surviving” haplotypes. Rows and columns of the vectors and matrices were rearranged to put equation 24 into the form

(25) $$\left[\begin{array}{l} q_{1}^{*A}\\ q_{2}^{*A} \end{array}\right]∼N\left(\left[\begin{array}{l} q_{1}^{*B}\\ q_{2}^{*B} \end{array}\right],\left[\begin{array}{ll} \text{var}{\left[q^{*A}\right]}_{11} & \text{var}{\left[q^{*A}\right]}_{12}\\ \text{var}{\left[q^{*A}\right]}_{21} & \text{var}{\left[q^{*A}\right]}_{22} \end{array}\right]\right)$$

The frequencies of the components of the vectors were renormalized, such that $q_{2i}^{*A}=q_{2i}^{*B}=0$, while

$$\underset{i}{\sum }q_{1i}^{*A}=\underset{i}{\sum }q_{1i}^{*B}=1$$

We obtain the result that the conditional distribution of $q_{1}^{*A}$ has the mean

(26) $$\mu =q_{1}^{*B}+\text{var}{\left[q^{*A}\right]}_{12}{\left(\text{var}{\left[q^{*A}\right]}_{22}\right)}^{-1}\left(-q_{2}^{*B}\right)$$

and covariance matrix

(27) $$\Sigma =\text{var}{\left[q^{*A}\right]}_{11}-\text{var}{\left[q^{*A}\right]}_{12}{\left(\text{var}{\left[q^{*A}\right]}_{22}\right)}^{-1}\text{ var}{\left[q^{*A}\right]}_{21}$$

Using these parameters to define a Gaussian distribution, we calculated the likelihood of a bottleneck *N^T^* given the data for the surviving haplotypes represented by $q_{1}^{*A}$.

To account for the haplotypes which became extinct during transmission, we made the assumption that these died out at the point of transmission to the founder population, the rapid growth of the founder population ensuring that no haplotypes went extinct through genetic drift, and viral sequencing of a large number of viral particles ensuring that no haplotypes were missed by the sequencing process. Under this assumption, the log likelihood of extinction is given by the simple binomial likelihood

(28) $$\log\left[{\left(1-\sum_{i}q_{2i}^{*B}\right)}^{N^{T}}\right]$$

Summing the log likelihoods calculated for the surviving and the extinct haplotypes gave the total likelihood of the bottleneck size *N^T^*; the maximum likelihood value was identified via a simple optimization process. To prevent nonsensical outcomes at very low bottleneck sizes, we further imposed the constraint that *N^T^* could not be less than the number of haplotypes which survived transmission.

### Explicit method for bottleneck estimation.

The explicit method uses the inferred haplotype frequencies for the population before transmission to reconstruct the space of possible outcomes in the recipient individual. Given our inferred haplotype frequencies $q_{i}^{B*}$, we assume that *N^T^* viruses are transmitted. The probability that the founding viral population includes *n_i_* copies of the haplotype *i*, where Σ_i_ *n_i_* = *N^T^*, is given by

(29) $$P\left(n_{1},n_{2}, . . .,n_{k}|q^{B*}\right)=\left(\begin{array}{c} N^{T}\\ n_{1}n_{2}. . .n_{k} \end{array}\right)\underset{i}{\prod }{\left(q_{i}^{B*}\right)}^{n_{i}}$$

where the first term in the right-hand side of the equation is the multinomial coefficient.

For each possible outcome {*n_i_*} of this multinomial process, we obtained an inference of the haplotype composition $\left\{q_{i}^{A}\right\}$ of the transmitted population given the relationship $q_{i}^{A}=n_{i}\text{/}N^{T}$ for each haplotype *i*. We then calculated the raw likelihood of observing the partial haplotype data collected posttransmission given this composition using the Dirichlet multinomial formulation described above, summing likelihoods over the possible outcomes of the initial transmission.

(30) $$\overset{}{\underset{\overset{n_{1}, . . .,n_{k}}{\underset{}{\sum }}n_{i}=N^{T}}{\sum }}P\left(n_{1},n_{2}, . . .,n_{k}|q^{B*},N^{T}\right)\left[\text{exp}\left(\underset{i}{\sum }\text{log }L_{D}\left(x_{i}^{A,P}|T_{i}q^{A},C\right)\right)\right]$$

In this way, we evaluate the likelihood of the bottleneck size *N^T^* given the inferred pretransmission haplotypes ***q****^B^* and the observed sequence data ***x****^A^*; this is in contrast to the compound method, which is based on ***q****^B^* and ***q****^A^*. We note that this approach neglects an explicit accounting for within-host growth of the population. Different assumptions about the dynamics of early viral infection can lead to changes in inferred bottleneck sizes (17); we are not confident that the biological reality of this phenomenon is well understood. Modifications to the Dirichlet multinomial distribution could potentially be used in this context; increasing the variance of the likelihood function would soften the effect of small changes in the underlying population.

This approach has both the advantage and the disadvantage of explicitly representing the full set of all possible multinomial outcomes of transmission. While in this sense it remains close to the biological reality, it rapidly becomes computationally expensive as the number of haplotypes *k* increases and as *N^T^* becomes large. For this reason, we propose it as being optimal for small values of *N^T^*.

We note that, in our application to data from a transmission study presented here, the case in which a high bottleneck was inferred involved very limited diversity within viral segments; this facilitated the application of this method to consider larger bottleneck sizes.

### Generation of simulated data.

Simulated data were generated using a simplified model of influenza transmission. Viruses were generated to have eight independent segments, of lengths equal to the segments of the A/H1N1 influenza virus. Each segment had five uniformly distributed polymorphic loci, making a theoretical total of 32 full haplotypes. Six haplotypes were chosen from this set under the constraint that each of the five loci had to remain polymorphic. The frequencies of these haplotypes were then randomly generated under the constraint of a minimum haplotype frequency of 5%, matching the parameters used in a previous study (17). We note that in the reconstruction of haplotypes, our code is likely not to identify very low-frequency haplotypes in the population due to the parsimony-driven approach.

Each transmission event was modeled as a simple multinomial draw, selecting a number of viruses equal to the bottleneck size from the donor population. Within-host growth was then modeled as a second multinomial draw, conferring a 22-fold increase in the population size (30). Partial haplotype data were generated from simulated short reads of each viral segment. Short reads with lengths derived from the data set of a recent influenza study (31) were generated (mean read length = 119.68, standard deviation [SD] read length = 136.88, mean gap length = 61.96, SD gap length = 104.48, total read depth = 102,825), with these reads being used to calculate the number of reads spanning each set of consecutive polymorphisms in each segment. Given these numbers, partial haplotype observations were generated using a Dirichlet multinomial sampling process.

An inference of the transmission bottleneck was carried out independently using simulated data from each viral segment. These inferences were then combined, summing the log likelihoods across different segments to obtain an overall maximum likelihood estimate. Within our simulated data, a small number of cases were identified in which the entire posttransmission population in a segment was inferred to comprise a haplotype that was not present above the cutoff frequency in the pretransmission population, equivalent to a case where a haplotype arose *de novo* in the population and swept to fixation before data could be collected. In such cases, data for the segment in question were ignored, calculating the transmission bottleneck across the remaining segments.

### Processing of sequence data.

Our method was applied to data from a recent study of influenza transmission among individuals in households (3). Data from transmission pairs identified in this study were aligned using the BWA software package (28) and then filtered using SAMFIRE (25) to remove reads with a median Phred score below 30 and to mask nucleotides with a Phred score below this value. Following the original study, sites in coding regions of the virus were then called at an allele frequency cutoff of 2%, following which reads were divided into sets of partial haplotype data.

Data describing the within-host evolution of influenza were used to evaluate the extent of noise in the data set. Noise in data arises both from the nonrepresentative sampling of viruses from the host and from the subsequent experimental steps used to generate sequence data (27); an overestimate of the extent of noise in data can lead to substantial errors in the inference of a transmission bottleneck (17). We here took a heuristic approach applied in a previous study (17). In a first step, data from all within-host single-locus trajectories were used to generate a provisional estimate of the extent of the noise in the data. Next, trajectories which under this estimate evolved in a manner consistent with selective neutrality were identified. Models of selective neutrality (constant allele frequency), constant selection (*dq/dt = sq*[*1 – q*]), and time-dependent selection (exact match to observed frequencies) were fitted to the data using the Dirichlet multinomial model of equation 4, requiring a difference of 10 units of BIC to favor the more complex model. Trajectories identified as neutral under this method were used to produce a final estimate of noise in the data; we inferred the parameter *C *= 660. Data from 43 putative transmission events were evaluated.

The estimate of an effective read depth for the case in which a very high bottleneck was inferred was conducted using SAMFIRE based upon allele frequency data and using a cutoff frequency for minority alleles of 2%.

### Availability of code and data.

Code used or generated during this project is available online, with Table S1, from https://github.com/cjri/VeTrans.
